# Investigating epidemiological distribution (temporality and intensity) of respiratory pathogens following COVID-19 de-escalation process in Catalonia, September 2016–June 2021: Analysis of regional surveillance data

**DOI:** 10.1371/journal.pone.0285892

**Published:** 2024-02-09

**Authors:** Víctor Guadalupe-Fernández, Erica Martínez-Solanas, Aurora Sabrià-Sunyé, Carol Ferrer-Mikoly, Ana Martínez-Mateo, Pilar Ciruela-Navas, Jacobo Mendioroz, Luca Basile, on behalf of The Epidemiological Surveillance Network of Catalonia

**Affiliations:** 1 Sub-Directorate General of Surveillance and Response to Public Health Emergencies, Public Health Agency of Catalonia, Generalitat of Catalonia, Barcelona, Spain; 2 Research Support Unit of Central Catalonia, University Institute for Research in Primary Health Care Jordi Gol i Gurina, Sant Fruitós de Bages, Spain; 3 CIBER Epidemiologia y Salud Pública (CIBERESP), Instituto Salud Carlos III, Madrid, Spain; University of Malaga: Universidad de Malaga, SPAIN

## Abstract

**Background:**

Following the low incidence rates of non-SARS-CoV-2 respiratory viruses registered during the strict lockdown enforced in the pandemic, a resurgence of several endemic viruses in Catalonia (Spain) was noted during the early summer of 2021.

**Objectives:**

In this study, we investigated whether the circulation of non-SARS-CoV-2 respiratory viruses in Catalonia, assessed by Microbiological Reporting System of Catalonia (MRSC) and the Epidemiological Surveillance Network of Catalonia, was affected by the strict lockdown measures, as well as, the implication of the Coronavirus Disease 19 (COVID-19) de-escalation process in the late season outbreaks registered during the 2020–2021 season.

**Study design:**

A retrospective comparison of epidemic patterns in the respiratory viruses’ incidence, using regional public health surveillance data from MRSC, was performed between weeks 26/2016 to week 27/2021. Data were expressed as the weekly total number of test positivity for individual viruses. A segmented negative binomial regression model was conducted, with two parameters included (level and trend) for each segment of the time series (2020 pre-lockdown, 2020 post-lockdown and 2021). Results were reported as a unit changed in the strict lockdown.

**Results:**

A total of 51588 confirmed cases of the different respiratory viruses were included in the analysis, the majority were influenza cases (63.7%). An immediate reduction in the weekly number of cases was observed in 2020 after the COVID-19 outbreak for human adenovirus virus (HAdV) (β2 = -2.606; P <0.01), human parainfluenza virus (HPIV) (β2 = -3.023; P <0.01), influenza virus (IFV) (β2 = -1.259; P <0.01), but not for respiratory syncytial virus (RSV), where the number of cases remained unchanged. During 2020, a significant negative trend was found for RSV (β3 = -0.170, P <0.01), and a positive trend for HAdV (β3 = 0.075, P <0.01). During 2021, a significant reduction in the weekly number of cases was also observed for all respiratory viruses, and a borderline non-significant reduction for HPIV (β3 = -0.027; P = 0.086). Moreover, significant positive trends were found for each viral pathogen, except for influenza during 2020–2021 season, where cases remained close to zero. The respiratory viruses increased activity and their late season epidemic start particularly affected children under 6 years old.

**Conclusions:**

Our data not only provides evidence that occurrence of different respiratory virus infections was affected by the strict lockdown taken against SARS-CoV-2 but it also shows a late resurgence of seasonal respiratory viruses’ cases during the 2020–2021 season following the relaxation of COVID-19-targeted non-pharmaceutical interventions.

## Introduction

Acute respiratory illness (ARI) is a major cause of morbidity in developed countries accounting for a large number of hospitalizations of especially young children [1–3]. Before the Coronavirus Disease 19 (COVID-19) pandemic, lower respiratory tract infection caused an estimated 2.7 million deaths annually worldwide, ranking fourth for all-cause mortality. Respiratory syncytial virus (RSV) and human parainfluenza virus (HPIV) are leading causes of lower respiratory tract infection, particularly in young children, accounting for over 1.6 and 0.2 of the hospital admissions in children younger than 5 years (per 1000 persons/year) [1, 4, 5]. In children younger than 2 years, RSV and HPIV often presents as a bronchiolitis and, in younger infants, it presents with nonspecific symptoms like apnoea and reduced oral intake. There is no RSV or HPIV vaccine and no population-level intervention exists beyond basic hygiene measures [2, 6]. In adults and elderly individuals, RSV and influenza virus (IFV) are a common cause of influenza-like illness (ILI) [7].

In response to COVID-19 pandemic, in March 2020 numerous non-pharmaceutical interventions (NPIs), including physical distancing measures, school closures, travel restrictions, and the wide use of facemasks, were adopted by the Spanish Government as a way to reduce transmission of the virus in the population [8, 9]. Accordingly, community mitigation strategies to prevent the spread of severe acute respiratory syndrome coronavirus 2 (SARS-CoV-2) revealed a drastic decline in medically attended ARIs and a decreased activity of seasonal respiratory viruses, particularly in IFV, RSV, human adenoviruses (HAdV) and HPIV [10, 11].

However, after relaxing containment measures once the COVID-19 pandemic had levelled off, some countries experienced a resurgence of seasonal respiratory virus infection cases, especially RSV infection was observed in the early summer of 2021 [12–16]. In June 2021, the Centres for Disease Control and Prevention (CDC) issued a health advisory to notify an increase of inter-seasonal RSV activity across parts of the Southern United States [16, 17]. What is more, in February 2021, the Department of Health of the Australian government had already reported an inter-seasonal resurgence of RSV infection in Australian children [14], which commonly affected in form of acute bronchiolitis.

Monitoring of COVID-19 at national and regional level have allowed assessing the impact of the pandemic, offering scientific evidence for decision-making regarding NPIs. At the same time, several countries have adapted and further developed their influenza surveillance systems to better describe the trends and identify changes in the spread of respiratory viruses, including the integration of the analytical results in future preparedness activities to prevent and control respiratory virus events.

According to the Ministry of Health of the government of Spain, unusual cumulative cases of respiratory viruses, particularly RSV and HPIV surged in June 2021later than the traditional seasonal peak of the common respiratory virus, following the easing of the COVID-19 regional restrictions. This has been a global trend amidst the COVID-19 pandemic [12–16, 18–22].

### Objective

The aim of the present study was to assess the impact of the gradual release of COVID-19-targeted restrictions on the epidemic spread of respiratory viruses such as IFV, RSV, HAdV and HPIV in Catalonia. Comparison of epidemic patterns in the respiratory viruses’ incidence between weeks 27/2020 to week 26/2021 compared with the average of those reported during the equivalent period of the four previous seasons (2016–2020).

## Material and methods

Sentinel epidemiological and laboratory-based virological surveillance plays a critical role in Catalonia’s Public Health Department for monitoring ARIs, particularly influenza and other respiratory viruses. The Public Health Agency of Catalonia has established the Microbiological Reporting System of Catalonia (MRSC) to improve monitoring and early responses to Public Health Emergencies. Since 1993, the MRSC has been responsible for the surveillance and evaluation of microorganisms relevant to public health. In 2015, in accordance with Decree 203/2015, the MRSC became a mandatory notification system for microbiologists who work in the public and private sector of the health-care system. The MRSC collects periodic updated information on microorganisms causing infectious diseases detected by laboratories and following diagnostic criteria [23]. The number of laboratories and centres participating in delivering the information on each microorganism to the MRSC varied during the study (S1 Table). Although, as mentioned earlier in the text, the notification of some specific microorganisms (e.g., IFV, RSV, HAdV and HPIV) is a legal requirement in Catalonia.

A descriptive retrospective study was carried out during five influenza seasons (from 27th June 2016 to 4th July 2021) for the presence of common respiratory pathogens in Catalonia, including influenza A, B and C virus, RSV, HPIV and HAdV. The study period was based on the influenza season, also known as flu season, which is defined as the months of the year when the activity of influenza viruses, along with winter seasonal viruses, increases significantly and normally ranges from week 40th (the end of September) until week 20th the end of May. The systematic collection of epidemiological and virological data from healthcare facilities and laboratories was made from week 27/2016 (2016/17 season) to week 26/2021 (2020/21 season) through the MRSC. Consequently, the stored data generated was extracted and retrospectively analysed.

Time trends in the weekly number of cases were applied to estimate changes in respiratory viruses’ incidence throughout the COVID-19 pandemic. These data were compared to the epidemiological distribution of positive results from the previous years. Missing data from healthcare facilities and laboratories, that did not send all information requested during the study period, was expected and not able to account for.

A suspected case ILI or ARI is defined as the sudden onset of symptoms with at least one of the following four systemic symptoms: fever or feverishness, malaise, headache, myalgia; and at least one of the following three respiratory symptoms: cough, sore throat, shortness of breath, according to the European Centre for Disease Prevention [24] and Control’s clinical criteria of ILI. Health care professionals collected biological samples from patients meeting the ILI or ARI case definitions at routine practice. ILI or ARI were examined using multiple laboratory tests: positive viral culture, polymerase chain reaction (RT-PCR) assays and antigen test, following diagnostic criteria [23].

The information collected from the MRSC included data on demographic, epidemiological and virological characteristics: age group (0–5; 6–14; >14 years old), sex (male/female), reporting date of microbiological diagnosis, reporting season of microbiological diagnosis (2016–2017, 2017–2018, 2018–2019, 2019–2020, 2020–2021) and viral microorganism agent identified (RSV, HPIV, HAdV and IFV). The age was calculated based on the difference between date of microbiological diagnosis and the date of birth. The reporting season ranges from epidemiological week 27th (the end of June) until epidemiological week 26th of the next year. Finally, a dummy variable was defined, strict lockdown (0, 1).

The main events and epidemiological measures implemented in Catalonia after the COVID-19 outbreak were reported in the supplementary material in S1 Fig.

## Microbiology

All specimens collected were examined using multiple automated and semi-automated platforms to detect human influenza (A, B and C viruses), HPIV, HAdV and RSV. Testing platforms (diagnostic kits and techniques) varied over time depending on the necessities of the population assigned, yearly budgetary availability, resources, and protocols followed by laboratories and centres, provided that they comply with the international standards and the following diagnostic criteria [23]. Specimens were tested with multiplex reverse transcription nested-polymerase chain reaction (RT-PCR) assay, the FilmArray Respiratory Panel (bioMérieux/ BioFire Diagnostics) [25, 26] that detects up to 20 organisms, Xpert Xpress SARS-CoV-2/Flu/RSV test (Cepheid, Sunnyvale, CA, USA) [27, 28], Allplex™ SARS-CoV-2/Flu A/Flu B/RSV (SC2FabR) kit (Seegene Inc., Seoul, South Korea) [29], cobas^®^ Liat^®^ Influenza A/B & RSVassay(Roche Diagnostics, Germany), MagNA Pure 96 system (Roche Diagnostics, Germany), Adenovirus ELITe MGB^®^ Kit (ELITechGroup, Inc., Bothell, WA).

### Statistical analysis

To analyse the effect of the COVID-19 outbreak in the respiratory viruses, an interrupted time series (ITS) analyses was performed. Due to the presence of over dispersion, a negative binomial regression model was used. The ITS design allowed to adjust for time trends, to estimate changes, as well as to account for autocorrelation. Moreover, it would evaluate the effect on the duration of the COVID-19 outbreak (for instance, whether the outbreak may only have an effect over the earlier weeks) and seasonal or cyclical effects.

In order to model the effect of the COVID-19 pandemic, two parameters (level and trend) were included for each segment of the time series (before and post-onset of the COVID-19 outbreak). The results and analysis were reviewed whether there were changes in level and trend that follow an intervention. Overall, a change in level constituted an immediate intervention effect, and a change in trend represented a gradual variation in the outcome.

For adenovirus, HPIV and RSV the following segmented regression model was used:

$${\text{Y}}_{\text{t}}=\;\beta_{0}+\;\beta_{1}\text{T}+\beta_{2}{\text{X}}_{\text{t}}+\beta_{3}{\text{TX}}_{\text{t}}$$

β_0_ represented the baseline level at T = 0, β_1_ was the change in the outcome associated with a time unit increase (representing the underlying pre-onset of the COVID-19 outbreak trend), β_2_ was the level change post-onset of the COVID-19 outbreak, and β_3_ indicated the slope change following the onset of the COVID-19 outbreak (using the interaction between time and intervention: TX_t_). Specifically, the time trends were divided into three categories, as different behaviour in viruses was observed in the descriptive analyses: *0*—before the COVID-19 outbreak, *1* –for year 2020 post-onset of the COVID-19 outbreak (from epidemiological week 11 onwards), and *2* –for year 2021 post-onset of the COVID-19 outbreak. The parameter of the slope change after the onset of the COVID-19 outbreak and it was also divided into two, according to year 2020 or 2021. A function of Fourier terms was included (two pairs of sine and cosine functions) to adjust for seasonality.

As the number of influenza cases, post-onset of the COVID-19 outbreak, was minimum (near zero), the same statistical model was no longer possible to reproduce, therefore a simplified model was used, which only account for the change in the level and not for the slope.

A sensitivity analysis was performed to confirm our main results. Firstly, the effect of the large-scale and strict lockdown imposed in the country was assessed to determine the impact on the notification of respiratory viruses’ cases. Here, a binary variable indicated the epidemiological weeks which were affected by the period of the pandemic (Before/After). Afterwards, the interaction between the COVID-19 incidence and the incidence level of the respiratory viruses was evaluated, by including the weekly number of COVID-19 cases in the model.

The level of significance was established at 5%, therefore a p-value < 0.05 was considered statistically significant. All analyses were performed with R software (version 4.0.3), with DHARMa and MASS packages.

### Ethical statement

This study used routine data from public health surveillance activities, as part of the legislated mandate of the Health Department of Catalonia, which is the competent authority for the surveillance of communicable diseases and officially authorized to receive, treat and temporarily store personal health data. Therefore, all study activities were part of the public surveillance and were, as a result, exempt from Ethical board review and did not require informed consent. All analyses were based on pseudonymised data according to the principles expressed in the Helsinki Declaration.

## Results

### Descriptive statistics

Between weeks 27/2016 and 26/2021, a total number of 51,588 confirmed cases of the different respiratory viruses were obtained and analysed, the majority were influenza cases (63.7%), followed by RSV, HAdV and HPIV (Tables 1 and 2). Weekly descriptive statistics of number of viruses reported in Catalonia by season were included in the S2 Table. There was a significant difference in sex among patients between seasons (annual mean of females, 5,890; 49% vs. females of 2020–2021 season, 1,694, 46.6%; p≤0.005), however, no significant differences were observed in sex among patients by aetiological agent. The median age from 2016–2020 season (42.6 years; [0–106]) was older than from 2020–2021 season (5.3; [0–97]). Specifically, compared with 2016–2020, the proportion of infants aged 0–2 increased significantly during 2020–2021 season (3,195; 26.7% vs. 2,640; 72.7%, p <0.001). In contrast, the proportion of patients aged >14 decreased significantly during 2020–2021 season (7,351; 61.3% vs. 125; 3.4%). Amongst the different respiratory viruses considered in this study, significant differences were observed between age groups from 2020–2021 and 2016–2020 seasons, except for IFV (N = 8,208 vs. N = 18, p ≥ 0.05), which relates in particular to the absence of positive cases during 2020–2021 season. In this respect, the proportion of respiratory viruses changed between seasons. IFV and RSV were the most commonly detected pathogens between 2016 and 2020, and RSV and HPIV were the most commonly detected pathogens between 2020 and 2021 (Table 1).

**Table 1:** Descriptive statistics of respiratory pathogens reported in Catalonia before and post-onset of the COVID-19 outbreak.

	Adenovirus (HAdV)						Influenza (IFV)						Parainfluenza Virus (HPIV)						Respiratory Syncytial Virus (RSV)					
	Before			After			Before			After			Before			After			Before			After		
	N	%	$\bar{\text{X}}$	N	%	*χ* ^2^	N	%	$\bar{\text{X}}$	N	%	*χ* ^2^	N	%	$\bar{\text{X}}$	N	%	*χ* ^2^	N	%	$\bar{\text{X}}$	N	%	*χ* ^2^
Sex																								
Female	904	45.3%	226	358	46.2%	0.695	16,368	49.8%	4,092	10	55.6%	0.804	578	45.6%	145	417	45.8%	0.959	5,711	48.1%	1,428	909	47.1%	0.422
Male	1,090	54.6%	273	416	53.7%		16,464	50.1%	4,116	8	44.4%		689	54.4%	172	493	54.2%		6,153	51.8%	1,538	1,020	52.9%	
Unknown	1	0.1%	0	1	0.1%		11	0.0%	3		0.0%			0.0%	0		0.0%		16	0.1%	4		0.0%	
Age			0						0						0						0			
0–2	1,261	63.24%	315	573	74.03%	<0.001	3,366	10.25%	842	2	11.11%	0.695	636	50.20%	159	581	63.85%	<0.001	7,516	63.35%	1,879	1,484	76.93%	<0.001
3–5	396	19.86%	99	141	18.22%		2,270	6.91%	568	2	11.11%		136	10.73%	34	220	24.18%		509	4.29%	127	340	17.63%	
6–14	179	8.98%	45	38	4.91%		2,006	6.11%	502	2	11.11%		89	7.02%	22	73	8.02%		188	1.58%	47	50	2.59%	
>14	158	7.92%	40	22	2.84%		25,190	76.72%	6,298	12	66.67%		406	32.04%	102	36	3.96%		3,651	30.77%	913	55	2.85%	
Total	1,994	100,0%	499	774	100.0%		32,8832	100.0%	8,208	18	100.0%		1,267	100.0%	317	910	100.0%		11.864	100.0%	2,966	1,929	100.0%	

***χ***^**2**^: Chi-square

$\bar{\text{X}}$: Annual mean

**Table 2:** Descriptive statistics of respiratory pathogens reported in Catalonia before and post-onset of the COVID-19 outbreak, continued.

	Total					
	Before			After		
	N	%	$\bar{\text{X}}$	N	%	*χ* ^2^
Sex						
Female	23,561	49.1%	5,890	1.694	46.6%	0.004
Male	24,396	50.8%	6,199	1,937	53.3%	
Unknown	28	0.1%	7	1	0.0%	
Age						
0–2	12,779	26.65%	3,195	2,640	72.71%	<0.001
3–5	3,311	6.90%	828	703	19.36%	
6–14	2,462	5.13%	616	163	4.49%	
>14	29,405	61.32%	7,351	125	3.44%	
Total	47,957	100.0%	11,989	3,631	100.0%	

***χ***^**2**^: Chi-square

$\bar{\text{X}}$: Annual mean

Coinfection was identified in 3.0% (1,326 of 44,448) of the patients of 2016–2020 and in 10.6% (427 of 3,920) of the patients of 2020–2021 (Table 3). A higher prevalence of pathogenic microorganism coinfection among RSV patients was identified in both season periods. However, it was observed that HPIV was the most common microorganism during 2020–2021. For mixed viral infection, it was found that type of double infection most frequent was RSV and IFV during the season period 2016–2020 and, during the season period 2020–2021, HAdV and HPIV was the most common combination.

**Table 3:** Respiratory virus co-infections before and post-onset of the COVID-19 outbreak.

Respiratory virus co-infections											
**Proportion co-infections Before**											
Patogen	N total	*Influenza*		*Adenovirus*		*Parainfluenza*		*Respiratory Syncytial Virus*		Total	
		N. Coinf	% Coinf	N. Coinf	% Coinf	N. Coinf	% Coinf	N. Coinf	% Coinf	N. Coinf	% Coinf
*Influenza*	32,353			84	4.5%	3	0.3%	362	3.5%	443	1.0%
*Adenovirus*	1,955	84	0.3%			56	4.9%	124	1.2%	259	0.6%
*Parainfluenza*	1,250	3	0.0%	56	3.0%			45	0.4%	99	0.2%
*Respiratory Syncytial Virus*	11,797	362	1.2%	124	6.7%	45	3.9%			525	1.2%
Total	47,355	443	1.4%	259	13.9%	99	8.6%	525	5.1%	1,326	3.0%
**Proportion co-infections After**											
	N total	*Influenza*		*Adenovirus*		*Parainfluenza*		*Respiratory Syncytial Virus*		Total	
		N. Coinf	% Coinf	N. Coinf	% Coinf	N. Coinf	% Coinf	N. Coinf	% Coinf	N. Coinf	% Coinf
*Influenza*	497			6	0.8%	6	0.6%	5	0.2%	7	0.2%
*Adenovirus*	813	6	35.3%			68	7.2%	43	2.0%	109	2.8%
*Parainfluenza*	927	6	35.3%	68	8.6%			65	3.0%	136	3.5%
*Respiratory Syncytial Virus*	1,996	5	29.4%	43	5.4%	65	6.9%			128	3.3%
*Covid-19*	678,678	1	5.9%	7	0.9%	11	1.2%	28	1.3%	47	1.2%
Total	4,233	7	41.2%	109	13.8%	136	14.4%	128	5.9%	427	10.9%

### Seasonal distribution

**Fig 1:**
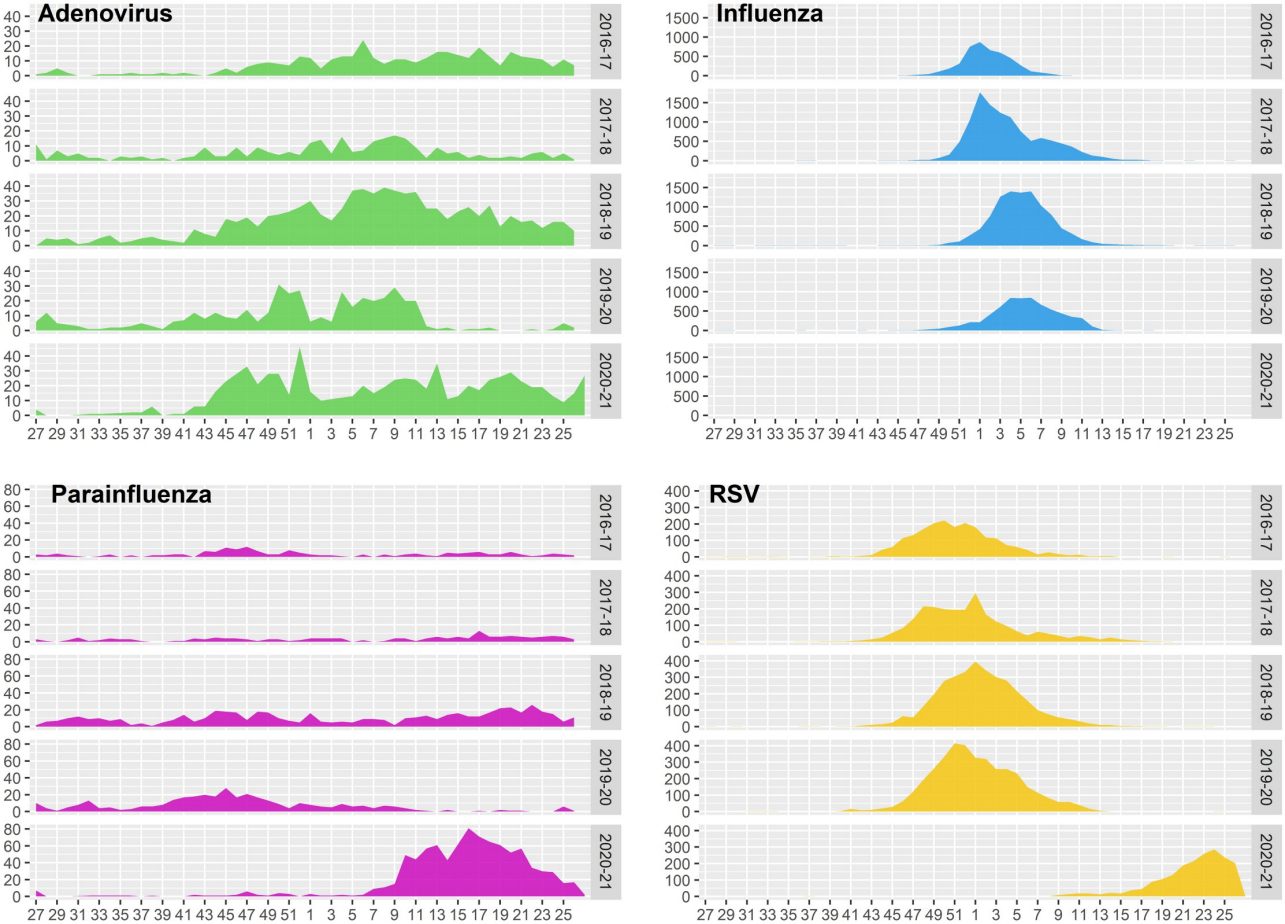
Seasonal activity of respiratory pathogens from season 2016–2017 to 2020–2021 in Catalonia. Weekly number of positive cases for each respiratory virus reported in Catalonia by season (2016–2017 to 2020–2021). Seasons range from week 27th (the end of June) until week 26th of the next year. RSV: Respiratory Syncytial Virus.

The results of the ITS models is shown in Table 4. An immediate reduction in the weekly number of cases was observed for each virus in 2020 for HAdV (β2 = -2.606; P <0.01), HPIV (β2 = -3.023; P <0.01), IFV (β2 = -1.259; P <0.01), but not for RSV, where the number of cases remained unchanged (Table 4). We observed that HAdV and HPIV developed a delay epidemic start, increasing activity late in the season. In this respect, a more intense epidemic curve may be observed at the end of the epidemic season (Fig 1). RSV activity showed a late introduction at the end of the season. IFV did not show any increased activity during 2020–2021 season.

**Table 4:** Interrupted time series analysis of weekly number of viruses reported in Catalonia before and post-onset of the COVID-19 outbreak.

	Adenovirus			Influenza			Parainfluenza			Respiratory Syncytial Virus		
	β	SE	P-value	β	SE	P-value	β	SE	P-value	β	SE	P-value
Constant (β0)	1.503	0.093	<0.01	2.275	0.167	<0.01	0.775	0.109	<0.01	1.836	0.097	<0.01
Time (β1)	0.006	0.001	<0.01	0.002	0.001	0.205	0.010	0.001	<0.01	0.003	0.001	<0.01
Level (β2)												
2020	-2.606	0.266	<0.01	-1.259	0.283	<0.01	-3.023	0.373	<0.01	-0.119	0.273	0.663
2021	-1.109	0.252	<0.01	-6.522	0.458	<0.01	-0.545	0.279	0.051	-6.879	0.320	<0.01
Trend (β3)												
2020	0.075	0.010	<0.01	-	-	-	0.017	0.015	0.250	-0.170	0.016	<0.01
2021	0.043	0.015	<0.01	-	-	-	0.048	0.016	<0.01	0.522	0.019	<0.01

Β: beta value

SE: Standard error

P-value: 5% cut-off for statistical significance

During 2020, a significant negative trend, a decreasing slope in the ITS, was found for RSV (β3 = -0.170; P <0.01), and a positive trend, an increasing slope in the ITS, for HAdV (β3 = 0.075; P<0.01) (Table 4). During 2021, a significant reduction in the weekly number of cases was also observed for all respiratory viruses, except for HPIV (β2 = -0.545; P = 0.051), with a borderline non-significant value. This effect was especially remarkable for IFV (β2 = -6.522; P <0.01) and RSV (β2 = -6.879; P <0.01). Moreover, significant positive trends were found for each viral pathogen, except for IFV, where cases remained close to zero. Sensitivity analysis when the strict lockdown was included in the model showed a less decline in the number of cases of viral pathogens during the year 2020 (S2 Table).

### Age-dependent distribution

**Fig 2:**
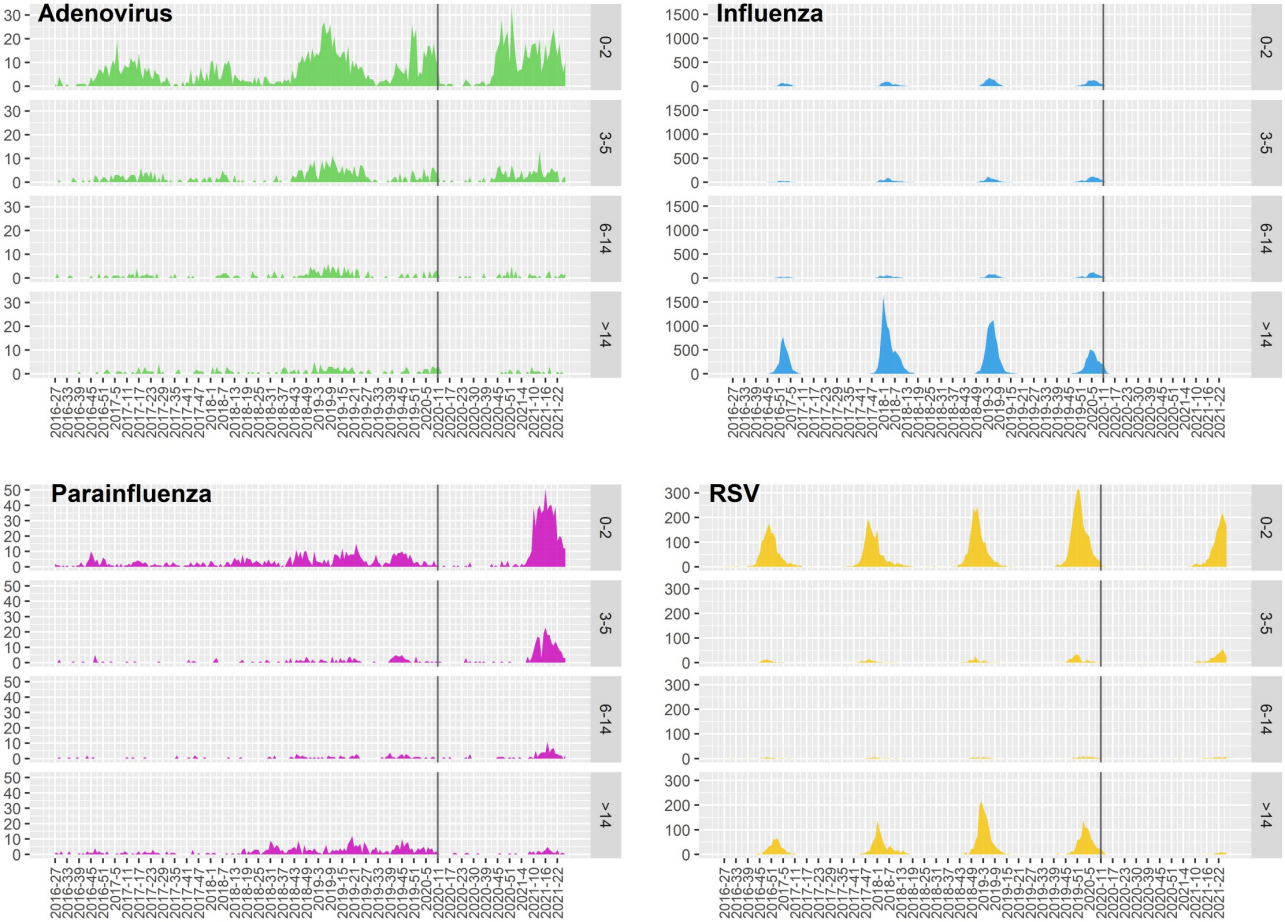
Age-dependent distribution of respiratory pathogens from season 2016–2017 to 2020–2021 in Catalonia. The weekly number of positive cases for each respiratory virus reported in Catalonia by age groups. The black line represents epidemiological week 11 of 2020. RSV: Respiratory Syncytial Virus.

On March 2020, there was a sharp drop in the number of cases notified in all age groups for all respiratory viruses analysed (Fig 2). After declining, the HPIV activity increased notably in the late season of 2020–2021, experiencing a shorter and an intense epidemic trend in children aged 0–2 and 3–5 years. The increased activity in the HPIV trend was less clear-cut for the 6 to 14 group and those over 14 years old. The late introduction of the typical seasonal distribution pattern of RSV was sharp for the age group of 0–2 years, but minor for the 3 to 5 group. Seasonal distribution patterns of IFV no longer existed after the lockdown in the different age groups. Increased activity of HAdV, with a longer epidemic curve, was experienced during 2020–2021 season, especially at younger ages (0–2 years and 3–5 years). Additionally, three consecutive waves were observed for children aged 0–2 years during this period. A dramatically lower detection levels of HAdV and RSV for individuals older than 6 years was observed during the 2020–2021 season compared to pre-pandemic levels.

## Discussion

The implementation of national lockdown to address the worldwide pandemic of COVID-19, followed by the COVID-19 de-escalation process, had an impact to the circulation of respiratory pathogens, as demonstrated by inter-seasonal RSV epidemics in several southern hemisphere countries [10, 14, 21] and late-season RSV and HPIV outbreaks in several European countries [12, 13, 15, 20, 30]. Since the beginning of the pandemic, many countries have observed a near total disappearance of respiratory viruses, especially RSV and influenza cases [11, 22, 31].

Overall, these results of this study suggested that, in Catalonia, the 2020 strict lockdown was the main initiator of the decrease in viruses’ circulation [11, 22, 30, 31]. However, the following autumnal, winter and spring lockdown, where curfews and less-restrictive measures were the main non-pharmaceutical interventions implemented for achieving control of the transmission of SARS-CoV-2, did not affect in the same way for each virus [16, 30, 32].

We observed that the seasonal distribution of flu disappeared after the declaration of the state of emergency by the government of Spain and the imposition of a nationwide lockdown on March 14th. The 2020–2021 flu season until week 26 remained near zero and the drop-off in influenza cases was experienced worldwide [11, 16, 32].

On the other hand, RSV became the most common virus during 2020–2021 season instead of IFV, such as over the last periods (2016–2020). According to the study of Weinberger Opek et al. [12], the spring/summer cases (75%) of 2021 were born at the beginning of March 2020, when RSV incidence rates decreased to zero, and they reached the spring/summer of 2021 with no previous exposure or development of immunity, coinciding with the relaxation of COVID-19-targeted non-pharmaceutical interventions. The seasonality of RSV in Catalonia changed during 2020–2021 season and had a 5-month delay, reaching its peak at mid-June. An out-of-season RSV epidemic was also shown in Southern Italy, beginning in August and peaking in November 2021 [3].

HAdV infections may occur anytime throughout the year, although they commonly peak in late winter, spring and early summer [33]. This study showed a 6-month absence of infections after the strict lockdown. The beginning of the epidemic curve overlapped the start of the school year throughout the first week of September (week 37). Most evidence points that young children are more sensitive and susceptible to HAdVs, due to lack of humoral immunity, particularly infants from age 6 months to 2 years of age who, as seen in our study, are also highly affected [34].

The circulation of HPIV shared similarities with HAdV, but remained absent for a longer period, until week 10 2021. It should be noted that parainfluenza resurgence had a shorter epidemic duration but bigger epidemic size, compared to the same period of the previous years. Our results suggested a reduction of herd immunity in children, resulting in a more intense epidemic size, especially in the first two years of life [10].

In contrast to the other studies, these results suggest a low rate of co-infection between SARS-CoV-2 and other respiratory pathogens [35, 36]. In line with our results, a recent systematic review and meta-analysis also showed a relatively low proportion of a respiratory viral co-infection in COVID-19 patients [37]. Accordingly, IFV and enteroviruses were the most prevalent pathogens in COVID-19 patients, which are among the most prevalent throughout the year, showing a higher prevalence of viral co-infection in the paediatric population and the elderly [37, 38]. A higher prevalence of bacterial co-infection comparing to viral co-infection have been recently described in COVID-19 patients [36]. Co-infection in hospitalized patients with ILI are a major cause of morbidity and mortality, however, some studies confirmed that viral-bacterial co-infection lead to more severe diseases than viral-viral co-infections [39, 40]. What is more, some studies pointed out improved outcomes in SARS-CoV-2 patients associated to viral co-infection [39, 41]. It is to be noted that in this study, routine testing for other respiratory pathogens when there was a positive result of SARS-CoV-2 infection was unlikely to happen, since there is no guarantee of providing benefits, unless the positive result for non-SARS-CoV-2 respiratory pathogens would change disease management [35].

Methods to evaluate virus competition have been previously assessed in vitro [42–44]. The theoretical feasibility of the competition between two or more viruses, as a possible explanation of the reduced circulation of IFV and other respiratory viruses during the COVID-19 pandemic has been explored as mentioned in some related studies [45–47]. However, according to surveillance data, evidence of the public health impact of such interaction has not been described yet during the COVID-19 pandemic. This might be an important contributor factor in the late winter resurgence of RSV and HPIV that should be addressed in the future. The different contagiousness and the higher percentage of asymptomatic patients in SARS-CoV-2 patients comparing to patients with of other respiratory viruses, such as IFV, could also affect in the way they spread in the population [48]. The delay in exposure to non-SARS-CoV-2 respiratory viruses and the potential increase of susceptibility in the population may be a plausible cause of the late season outbreaks experienced [10, 49]. In the paediatric population, their greater interaction in schools, immature immune system and the lack of used of faces masks, especially during the early summer, may have increased the opportunity of exposure to other less contagious respiratory viruses such as RSV and HPIV [38].

## Limitations

This study has its limitations. Firstly, the MRSC only has at its disposal the positive results of the analysed samples and reported from the laboratories; the negative results of the analysed samples are not reported, therefore, the mass or the volume of samples being analysed every year remains unknown. However, no appreciable differences regarding the number of laboratories reporting each microorganism were experienced during the study period, except for HPIV, where the number of reporting laboratories decreased after the COVID-19 pandemic. Even so, the increasing cases of HPIV cannot be explained for the aforementioned reasons, but if as a result of its increased circulation. Furthermore in this respect, it cannot be excluded that the decreasing trend of microorganisms may be related to the pressure exerted on the laboratories by the avalanche of COVID-19 test. Secondly, reports from laboratories and health centres were more likely to come from hospitalisations, especially severe cases, and general practitioners who are participants of the sentinels groups; therefore, unavoidable underreporting of cases were expected in this study. Thirdly, as mentioned earlier, the variety of testing platforms used among laboratories and health centres, the existing disparities in accessibility to some techniques or diagnostic kits among laboratories, due to the lack budgetary availability and the restricted and/or limited development of small hospitals and health centres, are the major causes of underreporting respiratory virus cases. Lastly, one of the main limitations of the study resides given the impossibility of addressing and comparing the data obtained with the weekly number of cases during subsequent seasons 2021–2022 and 2022–2023.

## Conclusions

Emergency response measures to reduce transmission of SARS-CoV-2 have also been effective in reducing the transmission of other endemic respiratory viruses in Catalonia. Data, from our ongoing surveillance system showed a late introduction of respiratory viruses during 2020–2021 season. Due to all non-pharmaceutical interventions (the strict lockdown and the implementation of hygienic and distancing measures), the burden of respiratory infectious diseases was drastically reduced. On the other hand, the immunity could have been negatively affected by the delay in the exposure to the virus due to the COVID-19 restrictions, with a potential increased of susceptibility in the population following the relaxation of COVID-19-targeted non-pharmaceutical interventions and causing late season outbreaks of non-SARS-CoV-2 respiratory pathogens, especially in the paediatric population.

The characterization of the changes in the circulation of respiratory viruses may help to shape public health recommendations to mitigate infections, especially in vulnerable populations, and to improve our understanding of the co-circulation of viruses. Respiratory pathogens surveillance should be reinforced during the pandemic and the de-escalation as COVID-19 restrictions become more flexible, in order to prevent any risk to the health of the susceptible population.

## Supporting information

S1 Fig Timeline of the COVID-19 pandemic in Catalonia, 2020–2022. (DOCX)

S1 Table Number of laboratories and centres that participate in the Microbiological Reporting System of Catalonia (MRSC). (XLSX)

S2 Table Weekly descriptive statistics of number of viruses reported in Catalonia before and after the COVID-19 outbreak. (XLSX)
