# Supplementary figures and images for: Investigating epidemiological distribution (temporality and intensity) of respiratory pathogens following COVID-19 de-escalation process in Catalonia, September 2016–June 2021: Analysis of regional surveillance data
Source: PLoS One. 2024 Feb 9;19(2):e0285892. doi: 10.1371/journal.pone.0285892 (PMC10857536; doi:10.1371/journal.pone.0285892)

**S1 Fig. Timeline of the COVID-19 pandemic in Catalonia, 2020-2022.**


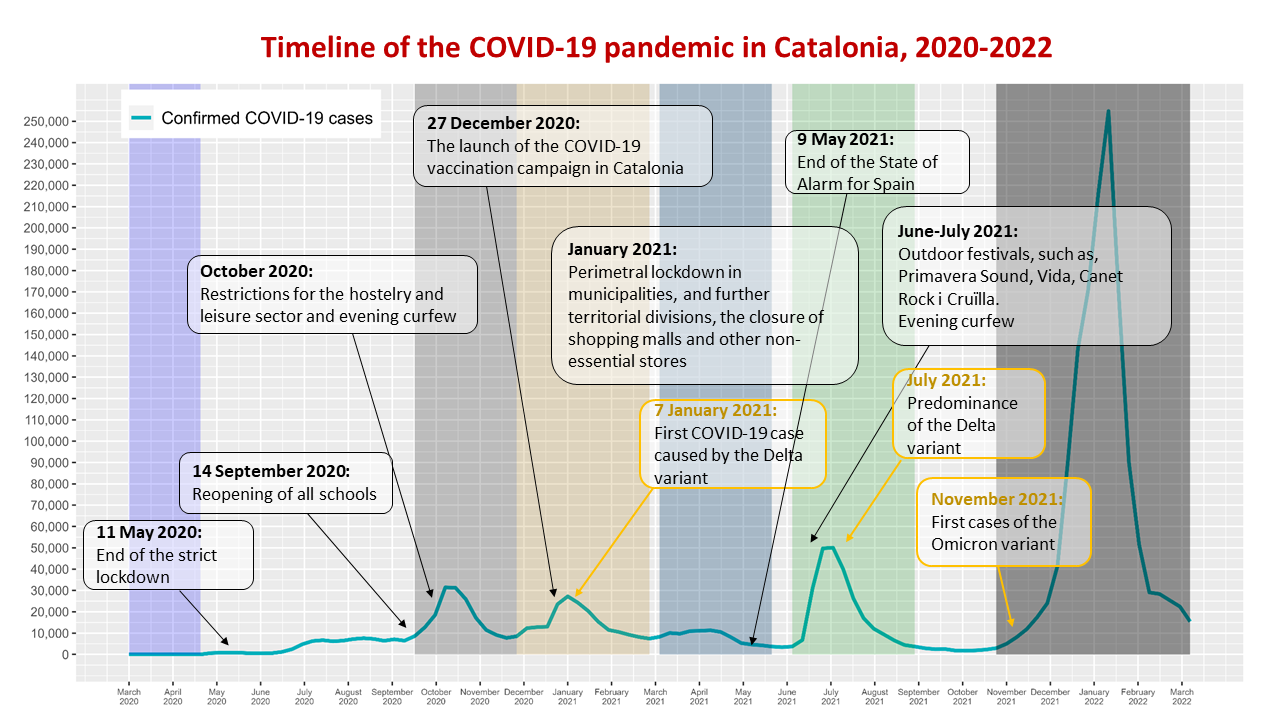

Supplement: S1 Fig — (DOCX) [file pone.0285892.s001.docx]
